# Comment on “The Effect of Chromium Picolinate Supplementation on the Pancreas and Macroangiopathy in Type II Diabetes Mellitus Rats”

**DOI:** 10.1155/2015/901895

**Published:** 2015-09-27

**Authors:** Hitesh Verma, Rajeev Garg

**Affiliations:** ^1^Overseas R&D Centre, Overseas HealthCare Pvt. Ltd., Phillaur, Punjab 144410, India; ^2^Department of Pharmaceutics, ASBASJSM College of Pharmacy, Bela, Ropar 140111, India

We read the research of Huang et al. [1] with great interest. Search of safe and efficacious alternative therapy for type II diabetes is always being the focus of research. Huang et al. explored the role of chromium in macrovascular complications of diabetes which has prime importance and is foremost leading cause of death in both type I and type II diabetic patients [2]. Present study have many appreciated results but interpretation of its results should be drawn with great caution. First and the foremost caution should be taken while escalating the dose of chromium picolinate. Huang et al. used three doses of chromium picolinate in their study (25 *μ*g/Kg, 50 *μ*g/Kg, and 100 *μ*g/Kg) but they did not make it clear whether these doses are in terms of elemental chromium or in terms of chromium picolinate salt. Based on previous research [3, 4] and the result obtained in Huang et al. study, probability there is that the mentioned doses are in terms of elemental chromium but in order to ascertain the fact we suggest Huang et al. to write corrigendum for present research. Since only then a correlation can be drawn between the previous research and their research work. Moreover, it will help future researchers in considering appropriate dose in clinical studies. Secondly, as macrovascular changes are most frequently associated with dyslipidemia, it would be of great help if one would add serum lipid parameters in future evaluation studies because levels of serum total cholesterol, triglycerides, low density lipoproteins, very low density lipoproteins, and high density lipoproteins are reported to have direct correlation with risk of developing macrovascular complications in type II diabetes [2]. Third, it is evident from the food consumption and weight variation data of animals that the present research is applicable to nonobese type II diabetic conditions. This is again in correlation with previous research in overweight and obese type II diabetic patients where even 1000 *μ*g/day dose of elemental chromium was unable to produce desired results [5]. We highly appreciate the work of Huang et al. in laying down the beginning stone of research related to the use of chromium in macrovascular complications of type II diabetes and will look forward for more future research work in this field.
